# Er,Cr:YSGG and 980nm diode lasers influence dentin surface volume after cariogenic challenge: *in vitro* study

**DOI:** 10.1590/1807-3107bor-2024.vol38.0045

**Published:** 2024-06-24

**Authors:** Fernanda Rodrigues Borges Amaral GUARATO, Marina Rodrigues SANTI, Isabela Ribeiro MADALENA, Vinícius Rangel Geraldo MARTINS, Maria Angélica Hueb de MENEZES-OLIVEIRA, Denise Tornavoi de CASTRO, Juliana Jendiroba FARAONI, Regina Guenka PALMA-DIBB, Cesar Penazzo LEPRI

**Affiliations:** (a) Universidade de Uberaba, Department of Biomaterials, School of Dentistry, Uberaba, MG, Brazil.; (b) Universidade Estadual de Campinas – Unicamp, Schoool of Dentistry of Piracicaba, Department of Restorative Dentistry, Piracicaba, SP, Brazil.; (c) Universidade de São Paulo – USP, School of Dentistry of Ribeirão Preto, Department of Restorative Dentistry, Ribeirão Preto, SP, Brazil.

**Keywords:** Lasers, Solid-State, Lasers, Semicondutor, Dentin

## Abstract

This study aimed to evaluate the influence of the Er,Cr:YSGG irradiation and 980-nm diode lasers on the surface roughness (SR) and volume loss (VL) of dentin subjected to cariogenic challenge. Subsequently, 130 specimens of bovine dentin were divided into the following 13 groups: NT: no treatment; FG: fluoride gel; FV: fluoride varnish; Di: 980-nm diode; Di + FG; Di + FV; FG + D; FV + Di; Er: Er,Cr:YSGG; Er + FG; Er + FV; FG + Er and FV + Er. Er,Cr:YSGG laser parameters were as follows: 0.25 W; 5.0 Hz; 4.46 J/cm^2^ without water and 55% air. Furthermore, the 980-nm diode laser parameters were 2.0 W; 2.0 Hz; 21.41 J/cm^2^. The samples from each group were subjected to pH cycling. A confocal laser scanning microscope was used to evaluate SR and VL. Difference between the volume of the reference and treated areas + DES/RE was used to determine SR and VL. The mean values of the different groups were subjected to analysis of variance and Tukey’s post-hoc test. The VL values were analyzed using the Kruskal–Wallis and Dunn post-hoc test (p < 0.05). The SR of the reference area did not show a statistically significant 1807-3107-bor-38-e025treatment and cariogenic challenge (p > 0.05). Moreover, VL in the FV + Di and FV + Er groups showed a statistically significant difference compared with areas submitted to different types of treatment and cariogenic challenge (p > 0.05). Er,Cr:YSGG and 980-nm diode lasers associated with fluoride varnishes decreased dentin VL in bovine teeth submitted to cariogenic challenge.

## Introduction

Erbium high-intensity lasers were pioneers in dentistry for cavity preparations, decayed tissue removal, and surgeries.^1-5^ Currently, the use of high-intensity lasers for preventive purposes has been widely researched.^3,6-8^ In recent years, evidence shows that irradiation with erbium (Er:YAG, Er:YSGG, and Er,Cr:YSGG) and diode lasers can promote an increase in the temperature of the irradiated surface, resulting in positive morphological and structural changes in the irradiated dental tissues.^1,2,4,6,8-12^

Increase in temperature caused by lasers in dental enamel can trigger chances and increase acid resistance, leading to the formation of fusion and recrystallization zones. Although there are controversies in the literature, studies suggest that dentin irradiation can increase mineral concentration through the preferential removal of water and proteins (collagen) inherent to this tissue, making it more resistant.^3,6,13^Moreover, formation of larger hydroxyapatite crystals than the original structure after laser irradiation on dentin may occur.^14-16^ Some studies showed that the association of Er,Cr:YSGG lasers with the application of fluoride and diode laser to dental tissues can generate beneficial effects in dentin due to increased acid resistance and fluoride retention on its surface.^4,10,13-15^The use of fluoride before laser application may lead the fluoride to be incorporated into the dentin, also known as fluoridated hydroxyapatite.^7^ Such additives also contribute to reducing surface roughness (SR) and dentin volume loss (VL), preventing the accumulation of dental biofilm, and consequently, reducing the risk/activity of dental caries.^4,10,13^

Since dental caries is the most common oral cavity disease,^17^ it is imperative to highlight its aesthetic–functional consequences and how it affects the quality of life of individuals.^18,19^ Thus, health promotion and prevention protocols have been extensively proposed. However, the change in tooth structure may limit the disease while the individual changes habits. The rate of root caries and difficulty in adherence of conventional restorative systems stand out.^20^ Regarding the use of lasers, scientific evidence is needed to define future preventive and therapeutic protocols that limit the spread of root caries. Thus, the present study aimed to evaluate the influence of Er,Cr:YSGG and 980-nm diode laser irradiation on SR and dentin VL in bovine teeth subjected to cariogenic challenge.

## Methods

### Aspect ethics

The study was conducted and reported following the Declaration of Helsinki and was approved by the Ethical Committee of the University of Uberaba (#028/2018).

### Experimental design

The study used 65 bovine tooth root samples. Bovine teeth were chosen because of their physical and mechanical similarity to human teeth.^21^ The bovine teeth were sectioned sagittally, originating in the mesial and distal halves. We prepared 130 specimens, which were randomly allocated to 13 different groups (n = 10; Table 1). The sample size was calculated considering a significance level of 5% and a test power of 85%. The quantitative variables were analysis of SR (μm^2^) and evaluation of VL (μm^3^).

**Table 1:** Experimental groups and treatment of specimens.

Groups	Treatment
NT	No treatment (control group)
FG	Fluoride gel
FV	Fluoride varnish
Di	980-nm diode laser
Di + FG	980-nm diode laser + fluoride gel
Di + FV	980-nm diode laser + fluoride varnish
FG + Di	Fluoride gel + 980-nm diode laser
FV + Di	Fluoride varnish + 980-nm diode laser
Er	Er,Cr:YSGG laser
Er + FG	Er,Cr:YSGG laser + fluoride gel
Er + FV	Er,Cr:YSGG laser + fluoride varnish
FG + Er	Fluoride gel + Er,Cr:YSGG laser
FV + Er	Fluoride varnish + Er,Cr:YSGG laser

### Selection of teeth

We selected 65 healthy bovine central incisors, and a single researcher used properly calibrated periodontal curettes to clean the bovine teeth. The remaining debris was refined with the Moto Emeril Tramontina 6” Bivolt 368 W equipment, using a circular brush in steel wire 0.3 mm, ensuring the removal of the entire cementum layer. Subsequently, the teeth were thoroughly washed and stored in distilled water at 4°C, with water being changed weekly.

### Preparation of the specimens

We used bovine teeth to simulate root caries. Subsequently, the crowns were separated from the roots 1 mm from the cement–enamel junction by using a diamond disc under water cooling coupled to a cutting machine. Before the second cut, the mesial and distal faces were identified with a small marking made with a spherical diamond-tipped drill FG 1013 (KG Brush, KG Sorensen, Cotia, Brazil) on the lateral face of the experimental area of all specimens. The second cut was performed using a precision cutting machine (ISOMET 1000^®^ cutting machine, Precision Saw Buehler, Waukegan Road Lake Bluff, USA), wherein the roots were cut sagittally, creating mesial and distal halves. The mesial and distal surfaces of the roots were chosen because of their flat surface and standardization of irradiation. The initial dimension of the specimens was 4.5 × 4.5 mm. Each specimen underwent wear in an APL metallographic sander and was polisher with #360 water sandpaper (Series 41042, Arotec SA industry and commerce, Cotia, Brazil), resulting in standardized blocks measuring 4.25 × 4.25 × 3.00 mm (final dimension), with a surface area of approximately 18.0 mm^2^ (9 mm^2^ experimental area and 9 mm^2^ control area); variations of 5% were allowed.

**Figure 1:**
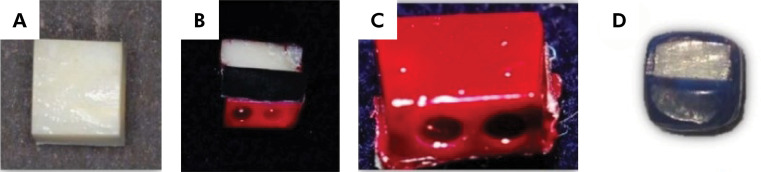
Preventive treatment of the specimens.

The experimental face was covered with insulating tape, whereas the control area and other faces were waterproofed with a double layer of cosmetic nail polish (Colorama Maybelline, São Paulo, Brazil). Except for the experimental half, a coating of sticky sculpting wax (Kota Industry e Commerce Ltda, Cotia,Brazil) was added to all sides after drying. Subsequently, the insulating tape was removed, resulting in an exposed dentin face for treatment and irradiation (Figure 1). The specimens were stored in distilled water at 4°C until treatment, wherein they were randomly divided into 13 groups, and each group received its treatment, as described in Table 1.

A. Standardized specimen. B. Specimen divided into control and experimental regions (insulating tape). C. Specimen with layers of nail polish. D. Specimen ready to receive treatment.

### Specimen treatment

Fluoride gel (1.23% acidified sodium fluoride, Nova DFL, Fluor Gel) was actively applied to the dentin surface with a microbrush (KG Brush, KG Sorensen, São Paulo, Brazil) for 4 min.^22^ Furthermore, fluoride varnish (5% sodium fluoride) Duraphat^®^ 22600 ppm fluoride (Colgate Palmolive Ind. e Com. Ltda, São Paulo, SP, Brazil) was actively applied to the dentin surface with a microbrush (KG Brush, KG Sorensen, CotiaBrazil). After 4 min, the excess was removed with gauze following the manufacturer’s recommendations. The fluoride varnish was left for 24 h, and the specimens were stored in water.^23^

### Laser parameters

An Er,Cr:YSGG laser (Biolase Technologies Inc., San Clemente, USA) was irradiated at a wavelength and a frequency of 2780 nm and 20 Hz, respectively. The laser was irradiated at 0.25 W at 1 mm. The groups where the Er,Cr:YSGG laser was applied before the gel or varnish (Er; Er + FG and Er + FV) were irradiated with 55% air. In contrast, in the groups where irradiation occurred simultaneously with the gel or varnish previously placed on the surface (FG + Er and FV + Er), irradiation occurred with no air/water system to avoid gel or varnish removal, although rinsing with water or drinking water does not affect the quantitative preventive effect (mineral content) of fluoride on the tooth.^24^ The total time of laser irradiation was 10 s for each sample. Subsequently, the samples were stored in distilled water in capped test tubes at 37°C.^7^

A 980-nm diode laser (DC International, Wellington, USA) was irradiated at 2.0 W and was in direct contact with the target tissue. Similarly, the total time of laser irradiation was 10 s for each sample.^7^

### Cariogenic challenge (pH cycling test)

Samples from each group were subjected to pH cycling to simulate a high caries risk condition. Each sample group was stored in its respective plastic container wherein the demineralizing (DE) and remineralizing (RE) solutions were added and changed. The corresponding containers/specimens were added with 50 mL of DE solution (2 mmol/L of calcium, 2 mmol/L of phosphate, and 75 mmol/L of acetate; pH = 4.6), and the samples were immersed for 6 h.^2^ Subsequently, the samples were removed, washed abundantly with distilled water for 10 s, and dried with gauze. The containers were also washed and dried. Meanwhile, the containers/specimens corresponding to the samples were added with 50 mL of the RE solution (1.5 mmol/L of calcium, 0.9 mmol/L of phosphate, 150 mmol/L of potassium chloride, and 20 mmol/L of cacodylate buffer; pH = 7.0), and the samples were immersed for 18 h. The RE solution has a grade of mineral saturation similar to saliva. DE and RE solutions were replaced daily (9:00 am and 3:00 pm, respectively; this cycle was carried out for 2 weeks. After 5 days, the samples were individually immersed in RE solution for 2 days (weekend), totaling a 14-day trial period. The samples were stored in a stove at 37°C during the trial period.

### SR and VL analyses

To analyze SR and VL, the specimens were positioned parallel to the table of the LEXT confocal laser scanning microscope (Olympus, Corp., Japan) with the aid of a parallelometer, performed by a single calibrated examiner. The central region (1 × 1 mm area) was selected and measured for these analyses, including the reference and treated areas + DE/RE. The images were obtained using a 20× magnification, generating a final magnification of 432×. Subsequently, these images were analyzed for SR (μm^2^ parameter) and VL. Data were acquired using specific software (OLS4100^®^). VL was determined by calculating the difference between the volume of the reference and treated areas + DE/RE between the midline of the specimen. The VL was obtained in μm^3^, and the data were transformed into VL percentages for statistical calculations.

### Statistical analysis

We used SPSS, version 17.1 for statistical analysis. Data followed a normal (Kolmogorov–Smirnov test) and homogeneous (Levene test) distribution. The mean SR values of the different groups were compared using parametric statistical analysis of variance. The Tukey post-hoc test was used to differentiate the means. The VL percentage values (%) were submitted to the Kruskal–Wallis nonparametric statistical test, followed by the Dunn post-hoc test. The significance level was 5% (α = 0.05) for all statistical tests.

## Results

Table 2 presents the SR results. The SR of the reference area showed no statistically significant difference when compared with areas subjected to different types of treatment and cariogenic challenge (p > 0.05).

**Table 2:** Surface roughness (µm2) mean values (standard deviation) of the groups, considering the reference area and the pre-treated area followed by the demineralization/remineralization cycles.

Groups	Reference area	Pre-treated area followed by DES/RE
NT	1.396 (0.109)^a^	7.940 (0.682)^c^
FG	1.395 (0.090)^a^	4.586 (0.322)^b^
FV	1.417 (0.080)^a^	4.528 (0.325)^b^
Di	1.340 (0.086)^a^	4.477 (0.360)^b^
Di + FG	1.421 (0.130)^a^	4.206 (0.424)^b^
Di + FV	1.352 (0.118)^a^	4.328 (0.409)^b^
FG + Di	1.456 (0.081)^a^	4.567 (0.340)^b^
FV + Di	1.439 (0.113)^a^	1.651 (0.149)^a^
Er	1.371 (0.106)^a^	4.257 (0.368)^b^
Er + FG	1.351 (0.127)^a^	4.467 (0.450)^b^
Er + FV	1.426 (0.073)^a^	4.411 (0.352)^b^
FG + Er	1.350 (0.088)^a^	4.390 (0.391)^b^
FV + Er	1.344 (0.065)^a^	1.523 (0.081)^a^

Equal letters represent statistical similarity between all lines and columns (p > 0.05).

**Figure 2:**
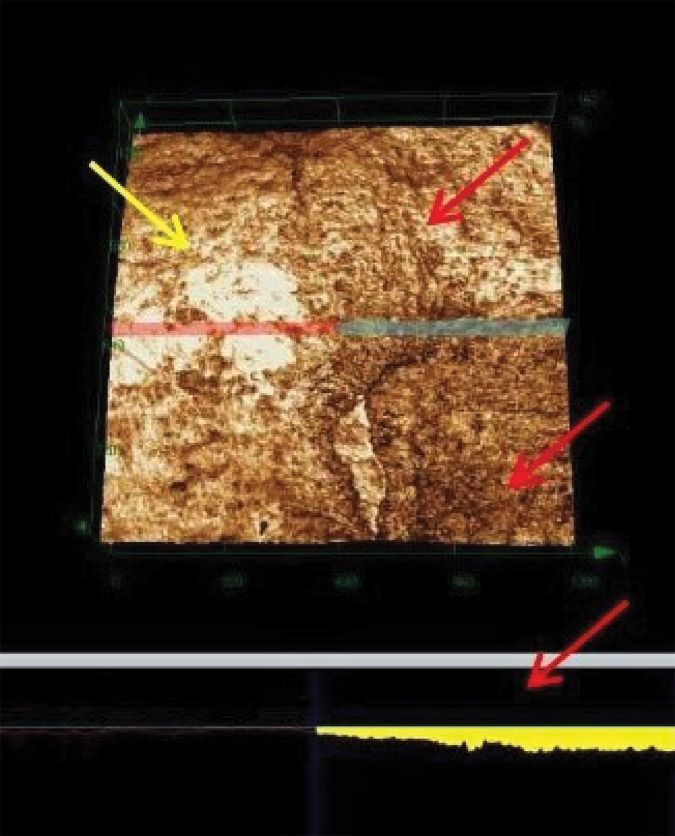
Volume loss between the experimental and control regions.

Regarding VL, all groups differed from the NT group; the FV + Di and FV + Er groups had lower VL percentages with a statistically significant difference between the other groups (p > 0.05; Table 3). Figure 2 shows the VL between the experimental and control regions.

**Table 3:** Volume loss mean values (standard deviation) of the groups.

Groups	Volume loss (%)
NT	58.9 (3.7)^e^
FG	46.1 (3.0)^d^
FV	34.2 (2.5)^c^
Di	20.7 (1.6)^b^
Di + FG	20.8 (1.7)^b^
Di + FV	19.7 (1.4)^b^
FG + Di	21.2 (2.1)^b^
FV + Di	13.5 (1.1)^a^
Er	18.5 (2.2)^b^
Er + FG	18.8 (2.0)^b^
Er + FV	17.8 (2.3)^b^
FG + Er	21.1 (1.9)^b^
FV + Er	11,6 (1,4)^a^

Equal letters represent statistical similarity between all lines and columns (p > 0.05).

Experimental (red arrow) and control regions (yellow arrow). Volume loss in the experimental area was also elucidated (red arrow).

## Discussion

Preventive laser irradiation, especially for the prevention of carious lesions, has been extensively studied.^3,4,6,7,11^ In recent years, Er:YAG lasers have been reported to cause morphological changes on the dentin surface that are superior to groups undergoing conventional preventive care, such as fluoride varnish, without significant thermal damage.^7^ Our results showed significant dentin VL in the groups subjected to conventional treatment or untreated. Thus, the null hypothesis was rejected.

Scientific evidence shows that the Er,Cr:YSGG laser reduces acid dissolution and mineral loss through chemical and morphological changes on the surface of dental tissues, making it positive for demineralization inhibition.^4,7,10,13^ Additionally, the Er,Cr:YSGG laser also promotes the dentinal tubule sealing through melting and solidification of dentin, which reduces the permeability of this tissue. Thus, this laser may be clinically useful to prevent dentin surface demineralization.^3,4,11,13^ Although our study results did not show statistical difference, Er,Cr:YSGG and 980-nm diode lasers promoted changes in SR and VL of the dentin structure, making it a less soluble tissue and more resistant to acid challenges, which was consistent with other literature reports.^3-7^ There were some limitations regarding sample size and applied energy density.

The present study showed specimen’s homogenization in the reference areas (reducing the chances of bias), noted by the low SDP value. The NT group showed the highest SR values, indicating that cariogenic challenge promotes greater damage to the dentin surface when untreated. The FG, FV, Di, Di + FG, Di + FV, FG + Di, Er, Er + FG, Er + FV, and FG + Er groups presented higher SR values compared with their respective reference areas; however, these values were lower than the control group, indicating that any treatment had better results than “no treatment.”^4,13,16^

Alhabdan et al. also reported increased SR of the irradiated areas^13^ and showed slightly rough surfaces depending on the energy density applied. For energy densities of 5.6 and 8.5 J/cm^2^, the study showed surfaces with shallow cavities, typical areas of microablation, conical fissures, and sharp enamel projections. However, the presence of globules of a highly agglomerated material similar to CaF_2_ can be observed after irradiation, showing a positive effect of the laser. Electron microscopy analysis also showed that the CaF_2_ globules formed after Er,Cr:YSGG laser irradiation (5.6 and 8.5 J/cm^2^) were greater than the globules formed in the fluoride gel group.^7^

Regarding surface volume, the groups with intermediate results (Di, Di + FG, Di + FV, FG + Di, Er, Er + FG, Er + FV and FG + Er) had lower VL compared with the NT, FG, and FV groups, indicating that the absence of treatment or the treatments with fluoride gel or fluoride varnish are inferior in relation to the irradiated groups or irradiated followed by fluoride groups. Fluoride, regardless of the form used, can superficially minimize the carious process. Furthermore, the groups receiving treatment with Er,Cr:YSGG and 980-nm diode lasers (Di and Er) alone were not statistically different in relation to the groups receiving treatments, neither with Er,Cr:YSGG and 980-nm diode laser previously to the application of fluoride (Di + FG, Di + FV, Er + FG and Er + FV) nor the groups receiving the previous application of fluoride gel (FG + Di and FG + Er) and subsequent irradiation with laser, demonstrating that irradiation alone with fluoride gel had no significant difference, which was consistent with studies that related the synergism between laser irradiation with the previous application of fluoride, mainly in high concentrations, in the form of fluoride varnish.^3-7,10,16^

The most promising results were observed in the FV + Di and FV + Er groups. Previous studies have demonstrated that laser irradiation leads to an increase in temperature on the dentin surface, thus melting hydroxyapatite structures. These structures, after cooling, makes dentin a tissue with a vitrified, non-porous surface having more occluded dentinal tubules areas.^1,4,6,15,16,25-30^Overall, the present study demonstrated that the groups that received previous treatment with fluoride had better results than the others. This can be explained by the possible incorporation of fluoride ions on the dentin surface during its melting and recrystallization.^9,28^This shows that the use of laser potentiates the effects of fluoride application, especially when laser irradiation is performed after fluoride application. This treatment sequence seems to be the most promising technique for dental caries prevention because fluoride ions are incorporated in the dentin structure, making it a tissue with more mineral content and more resistant.^1,2,4,10,11,26^ These theories concur with the recent study by dos Santos Ferreira et al.,^10^ who demonstrated that APF treatment before laser irradiation promoted chemical and morphological changes in dentin by significantly reducing the proportion of calcium carbonate, indicating collagen destruction of the dentin due to increased temperature. Furthermore, morphological results and dentin composition analyses showed a better thermal effect of laser irradiation after APF gel application.

The best results of our study were found in FV + Di and FV + Er, demonstrating that the previous application of fluoride varnish and subsequent irradiation with Er,Cr:YSGG and 980-nm diode lasers resulted in less dentin VL and SR, making them superior to the other groups. This superiority of treatment is related to the amount of fluoride available in the fluoride varnish (22,600 ppm of fluoride) because higher amounts of available fluorides result in better incorporation into the dentin surface.^31^ This theory is consistent with the study by Nóbrega et al.,^32^ who reported that higher amounts of fluoride (to achieve beneficial results) are required when the substrate is dentin. Additionally, the best results obtained with fluoride varnish were because of laser photoabsorption caused by the pigment present in the varnish, which increases photochemical reactions and laser absorption on the irradiated surface.^9^

The study showed that both lasers were effective, with no statistically significant differences, indicating the extremely promising effect of the 980-nm diode and its undeniable clinical applicability, given that it is a laser with low acquisition and maintenance costs as well as having greater versatility (compact equipment) in relation to the Er,Cr:YSGG laser. Therefore, the present study suggests that the association of fluoride plus irradiation with Er,Cr:YSGG or 980-nm diode lasers in pre-established parameters is a promising treatment protocol, with fluoride varnish being the best choice of fluorotherapy.

## Conclusion

Er,Cr:YSGG and 980-nm diode lasers associated with fluoride varnishes decrease dentin root VL in bovine teeth subjected to cariogenic challenge.
